# Gallic acid inhibits osteoclastogenesis and prevents ovariectomy-induced bone loss

**DOI:** 10.3389/fendo.2022.963237

**Published:** 2022-12-19

**Authors:** Peng Zhang, Jiekai Ye, Jiale Dai, Ying Wang, Genjun Chen, Jinping Hu, Qimiao Hu, Jun Fei

**Affiliations:** ^1^ Zhejiang Hospital of Integrated Traditional Chinese and Western Medicine, Hangzhou, China; ^2^ The Third Clinical College of Zhejiang Chinese Medical University, Hangzhou, China

**Keywords:** osteoclast, Gallic acid, Akt, ERK, JNK, osteoporosis

## Abstract

Osteoporosis is a common metabolic bone disease with a rapidly increasing prevalence, characterized by massive bone loss because of excessive osteoclast formation. Gallic acid (GA), a phenolic acid isolated from *Cornus officinalis*, has anti-inflammatory and anti-oxidant effects, but its effect on osteoclast formation has not been confirmed. In our study, we demonstrated that GA significantly inhibited RANKL‐induced osteoclast formation and function of osteoclast in bone marrow monocytes (BMMs) and RAW264.7 cells in a dose-dependent manner without cytotoxicity. For molecular mechanisms, GA repressed osteoclastogenesis by blocking Akt, ERK, and JNK pathways, and suppressed osteoclastogenesis-related marker expression, including nuclear factor of the activated T-cell cytoplasmic 1 (NFATc1), c‐Fos, and cathepsin K (CTSK). In addition, we further assessed the effect of GA in an ovariectomized mouse model, which indicated that GA has a notable effect on preventing bone loss. In conclusion, GA exerts notable effects in inhibiting osteoclastogenesis and preventing ovariectomy-induced bone loss, suggesting that GA is a potential agent in osteoporosis treatment.

## Introduction

Osteoporosis is a chronic metabolic bone disease commonly seen in postmenopausal women, characterized by massive bone loss because of excessive osteoclast formation, as well as increasing risk of fragility fractures (1). According to the report, about 40% of postmenopausal women over the age of 50 suffer from osteoporosis (2). The pathophysiology of osteoporosis is the disequilibration of bone homeostasis between osteoblast and osteoclast (OC) (3). Currently, therapeutic drugs for osteoporosis, such as bisphosphonates, calcitonin, and estrogen, increase bone mineral density and reduce the risk of fragility fractures (4). However, these pharmacological drugs for osteoporosis are still limited due to several side effects, including breast cancer, atypical femoral fractures, and hypercalcemia (5, 6). Therefore, it is crucial to explore safer and effective drug to regulate OC differentiation and function. Natural compounds have been reported for the treatment of osteoporosis due to their extensive bioactivities and limited side effects (7, 8), which provides us with a reliable basis to explore this field.

OC, derived from bone marrow monocytes (BMMs), are responsible for bone resorption (9). During OC differentiation, the two critical cytokines for OC formation and maturation are receptor activator of nuclear factor κ‐B ligand (RANKL) and macrophage colony‐stimulating factor (M‐CSF) (10). M‐CSF was considered the first essential factor to maintain the proliferation and vitality of preosteoclast cells and to stimulate the expression of receptor activator of nuclear factor-κB (RANK) (11). Meanwhile, RANKL plays an important role in OC differentiation by interacting with its receptor RANK (12). Once RANKL combines with its receptor RANK, then activates a series of downstream signaling pathways, and eventually result in enhanced transcription of c-Fos and NFATc1 (13, 14). Thereafter, these signaling cascades promote the expression of various osteoclastic genes and proteins including tartrate resistant acid phosphatase (TRAP) and cathepsin K (CTSK) (15).

Gallic acid (GA), a phenolic acid isolated from *Cornus officinalis*, exhibits extensive pharmacological activities, including anti‐inflammatory and antioxidant activities (16). It has been reported that GA exerts immunomodulatory effects on LPS-stimulated RAW264.7 cells by inhibiting MAPK, NF-κB and AP-1 pathways (17). In addition, GA also inhibits fibroblast growth and migration in keloids through suppressing the AKT/ERK signaling pathway (18). However, the effects of GA on OC differentiation and function have not yet been reported. Since the process of osteoclastogenesis shares these pathways, we investigated GA‐mediated regulation of RANKL‐induced OC formation from BMMs and RAW264.7 cells and its underlying mechanism.

Herein, we assessed the effects of GA on OC differentiation and function *in vitro and in vivo*, and further elucidated its mechanisms.

## Materials and methods

### Materials and reagents

GA (PubChem CID: 370) was supplied from FeiyuBio (Nantong, China) and dissolved into a storage concentration of 100mM with dimethyl sulfoxide (DMSO). FBS and α-MEM were purchased from Gibco (Rockville, MD, United States) and AusGeneX (Brisbane, Australia), respectively. Recombinant RANKL and M-CSF were provided by R&D (R&D Systems, MN, United States). Cell Counting Kit-8 (CCK-8) was supplied by Absin (Shanghai, China). Rhodamine‐conjugated phalloidin were purchased from Thermo (San Jose, CA, USA). The Cell Signaling Technology (Beverly, MA, USA) supplied the primary antibodies against p-AKT, AKT, p-ERK, p-JNK, JNK, p‐P38, and P38. The antibodies against β-Actin, NFATc1, Cathepsin K (CTSK), and ERK were obtained from Santa Cruz (San Jose, CA, USA). C-Fos were obtained from Abcam (Cambridge, MA, USA). RAW264.7 cells were kindly provided by Stem Cell Bank, Chinese Academy of Sciences. Sterile bone slices were obtained from IDS (London, UK).

### Cell culture

BMMs were extracted from the bone marrow cavities of tibias and femur of six-week-old mice and then cultured in α‐MEM medium with 25 ng/mL M‐CSF. After 48 h of culture, the suspended cells were discarded, and the remaining adherent cells were BMMs. In addition, RAW264.7 was incubated with α‐MEM medium without M‐CSF. Then, BMMs and RAW264.7 were all seeded for osteoclastogenic differentiation induction.

### Cytotoxicity assay

To test the cytotoxicity of GA on BMMs and RAW264.7, a CCK-8 assay was conducted according to the manufacturer’s protocol. BMMs (1 × 10^4^/well) and RAW264.7 (5 × 10^3^/well) were seeded into plates (96-well plates) and cultured separately overnight. Next, the cells were treated with various concentrations of GA (0, 1, 2.5, 5, and 10 μM) for 48 h, and then 10 μl CCK8 solution per well was added and incubated for another 3 h. At last, the absorbance at 490 nm was recorded using a multimode reader (Spark 10M, TECAN, Switzerland).

### Osteoclastogenesis assay

At a temperature of 37°C and 5% CO2 condition, BMMs (1 × 10^4^/well) and RAW264.7 (5 × 10^3^/well) were seeded into a plate (96-well plate) and cultured separately overnight to adhere. For osteoclastogenesis, adherent cells of BMMs and RAW264.7 were incubated with RANKL (50 ng/ml) and increasing concentrations of GA (1, 2.5, 5, and 10 μM) for 6 days (medium of each well replaced every 2 days). At day 7, fixed BMMs and RAW264.7 cells were performed for TRAP staining, followed by quantification of TRAP-positive multinucleated cells.

### Immunofluorescence staining

As mentioned above, BMMs (1 × 10^4^/well) were incubated overnight in the presence of 25 ng/mL M-CSF. After that, BMMs were induced into OC with RANKL (50 ng/mL) stimulation in the presence or absence of GA (5 μM, 10 μM) for 6 days. At day 7, the fixed cells were incubated with 0.5% Triton X-100, and then stained with rhodamine‐conjugated phalloidin. F‐actin were visualized under fluorescence microscope (Axioskop 40, Zeiss, Germany).

### Pit formation assay

The sterile bone slices (DT-1BON1000-96) were used to detect the function of OC. BMMs (1×10^4^/well) were seeded onto the sterile bone slices that had been pre-placed on the bottom of a 96-well plate and cultured overnight. Next day, RANKL (50 ng/ml) was added to stimulate OC differentiation in the presence or absence of GA (5 μM, 10 μM). The medium in each well was replaced every 2 days, and the cells were induced and cultured for 10 days. At day 11, all the bone slices were detected by scanning electron microscopy to observe the bone resorption pits. Finally, the bone resorption pits on every bone slice were measured by ImageJ.

### Quantitative real time PCR

In the presence of RANKL (50 ng/mL) and M‐CSF (25 ng/mL), BMMs (1×10^5^/well) were cultured in a 6‐well plate and treated with different concentrations of GA (5, 10 μM) for 6 days. Subsequently, total RNA was extracted and synthesized into cDNA with a cDNA Synthesis Kit (Bio-Rad, CA, USA). After that, the synthesized cDNA was used to perform real‐time PCR assay with a green RTPCR kit (Bio-Rad, CA, USA). The specific primers were displayed in **Table 1**. The expression of target genes was normalized to β-actin, which was used as control.

**Table 1:** Primer sequences used in qRT-PCR.

Genes	Sense sequence (5’-3’)	Antisense sequence (5’-3’)
NFATc1	GGAGAGTCCGAGAATCGAGAT	TTGCAGCTAGGAAGTACGTCT
c-Fos	GCGAGCAACTGAGAAGAC	TTGAAACCCGAGAACATC
CTSK	TGTATAACGCCACGGCAAA	GGTTCACATTATCACGGTCACA
β-actin	GGCTGTATTCCCCTCCATCG	CCAGTTGGTAACAATGCCATGT

### Western blot

To evaluate the long-term action of GA on the expression of NFATc1 pathway and OC function-related proteins, BMMs (1 × 10^5^/well) in a 6‐well plate were cultured and stimulated with RANKL (50 ng/mL) in the presence or absence of GA (5, 10 μM) for 6 days. Next, total protein was harvested using RIPA buffer to lyse the cells at day 6. Meanwhile, to assess the effect of GA on the expression of downstream signaling pathways in a short period of time, BMMs (5 × 10^5^/well) in a 6‐well plate were incubated with M‐CSF (25 ng/mL) overnight to adhere. Next day, the adherent cells were starved for 4 h and incubated with GA (5, 10 μM) for 2 h. Thereafter, RANKL (50 ng/ml) was added to stimulate OC differentiation for 1 h, and then total protein was lysed and obtained by using RIPA buffer.

The extracted proteins were loaded and separated by 10% SDS-PAGE gel, transferred to PVDF membranes, and blocked in 5% skim milk for 1 h and eventually incubated with primary antibodies against P38 (diluted 1:1000, #9212), p-P38 (diluted 1:1000, #4511), JNK (diluted 1:1000, #9252), p-JNK (diluted 1:1000, #4668), p-ERK (diluted 1:1000, #4370), p-AKT (diluted 1:1000, #4060), AKT (diluted 1:1000, #4691), ERK(diluted 1:1000, sc-514302), CTSK (diluted 1:1000, sc-48353), NFATc1 (diluted 1:1000, sc-7294), c-Fos (diluted 1:1000, ab222699), and β-Actin (diluted 1:10000, sc-47778) overnight at 4°C. Later, the PVDF membranes were subjected to incubation with secondary antibodies for 1 h at room temperature. Finally, the immunoreactive bands were detected with an Image Quant LAS 4000 system (GE, United States), followed by analysis using by ImageJ.

### Ovariectomy mouse model establishment

Ten-week-old female C57/BL6 mice, purchased from the animal center of Zhejiang Chinese Medical University (Hangzhou, Zhejiang), were randomly divided into the sham group (n=7), the OVX group (n=7), and the OVX treated with GA (OVX+GA) group (n=7). The mice in the sham group received intraperitoneal DMSO injection, while the ovariectomized mice in OVX and OVX + GA groups were intraperitoneally injected DMSO and 10 mg/kg GA every 2 days for 8 weeks, respectively. Based on previous similar studies, the dosage of intraperitoneal injection in this study was determined (7, 8). After 8 weeks of drug administration, the mice of all groups were sacrificed, and the bilateral femurs were obtained and subsequently used to histopathology analysis and Micro-CT scans. Animal care and experimental procedures were approved by the Ethics of Animal Experiments of Zhejiang Chinese Medical University (IACUC-20210913-06).

### Micro‐CT scanning of femurs

The isolated femurs from each group were scanned with a Skyscan 1176 micro-CT equipment (Bruker, Kontich, Belgium) at a resolution of 10 μm. Then, NRecon were used to reconstruct three-dimensional image, followed by analysis with CTAn software. Morphometric analysis of the following parameters was performed as our previous study (19): bone mineral density (BMD); trabecular bone volume per tissue volume (BV/TV); trabecular thickness (Tb. Th); trabecular number (Tb. N); trabecular separation (Tb. Sp); cortical thickness (Ct.Th).

### Histology and immunohistochemistry

Briefly, the femurs were fixed and decalcified, and then embedded in paraffin. Thereafter, femur tissues were cut to produce 4‐μm‐thick sections and subsequently subjected to Alcian Blue Hematoxylin/Orange G (ABH) and TRAP staining for morphological analysis and observation of OC formation. The area of lipid droplets, trabecular area, OC number per bone surface (N.Oc/BS) and the OC surface per bone surface (Oc.S/BS) in the region of interest (ROI) were measured by two researchers.

The steps of IHC assay are as follows: First, the blank sections were incubated in 0.01M citrate buffer (60°C, 4 h) and inactivated by H_2_O_2_ (37°C, 20 min). Subsequently, sections were subjected to incubation at 4°C with primary antibodies against CTSK (diluted 1:400, sc-48353), p-ERK (diluted 1:200, #4370), p-AKT (diluted 1:200, #4060), and p-JNK (diluted 1:200, #4668) overnight. Next day, DAB solution was applied and counterstained with hematoxylin. Finally, the quantification of positive staining in five consecutive sections was calculated using ImageJ software.

### Statistical analysis

All the data were presented as means ± SD. Analyses of our present research were conducted using one-way ANOVA test or student’s *t*-test with GraphPad Prism. *P*<0.05 was considered statistically significant.

## Results

### GA inhibits RANKL-induced osteoclastogenesis *in vitro*

**Figure 1:**
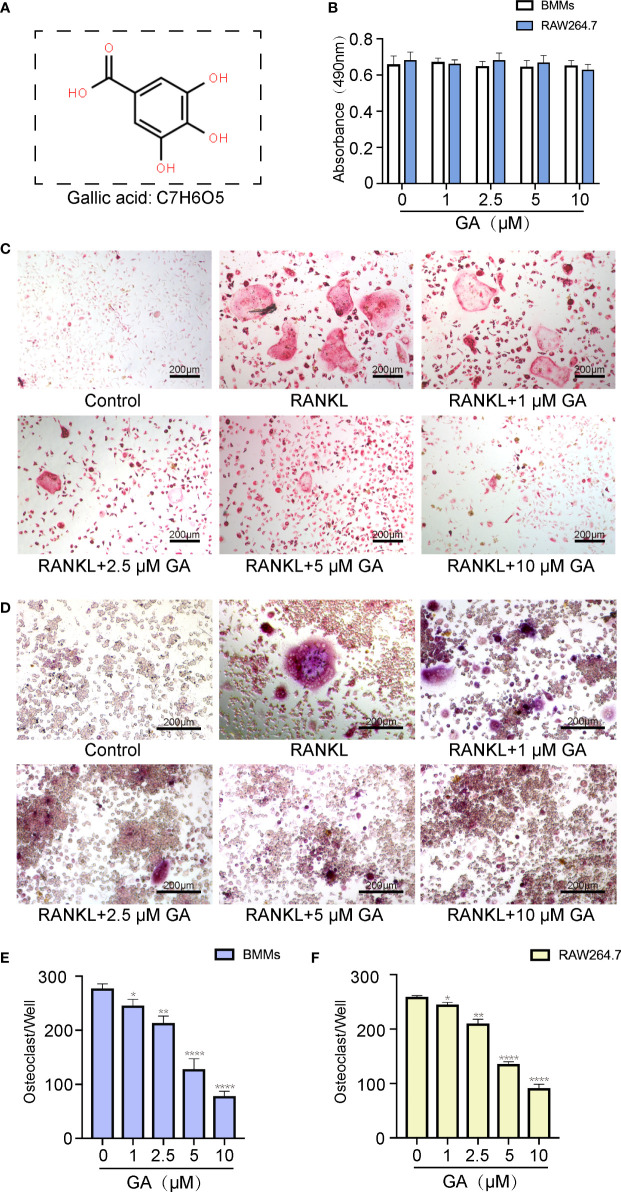
GA inhibits RANKL-induced osteoclastogenesis *in vitro.* **(A)** Chemical structure of GA. **(B)** CCK-8 assay was performed to measure the effects of the increasing concentrations of GA on the proliferation of BMMs and RAW264.7 cells. **(C, D)** TRAP staining of OCs induced from BMMs and RAW264.7 cells with increasing concentrations of GA. **(E, F)** Quantitative analysis of the TRAP-positive multinucleated OCs (>3 nuclei) induced from BMMs and RAW264.7 cells in each well. Scale bar = 200 μm. n= 5, *P < 0.05, **P < 0.01, and ****P < 0.0001 versus RANKL-induced group.

Before assessing the inhibitory effect of GA on osteoclast formation, we investigated its cytotoxicity against BMMs and RAW264.7 cells by CCK-8 assay (**Figure 1A, B**). The CCK-8 assay results indicated that GA exerted no significant toxicological effect on the proliferation of BMMs and RAW264.7 cells with increasing concentrations, which confirmed that the inhibitory effect of GA on osteoclastogenesis was not due to cell cytotoxicity.

To evaluate the suppressive effect of GA on OC formation, BMMs and RAW264.7 cells were induced into OC by RANKL (50 ng/mL) stimulation in the presence or absence of GA (1, 2.5, 5, and 10 μM). The results of TRAP staining indicated that the suppressive effect of GA on OC formation is clearly dose-dependent (**Figures 1C, D**). Moreover, the number and size of TRAP^+^ OC cells in each well of 96-well plate were dose-dependently reduced at concentrations of GA higher than 2.5 μM (**Figures 1C–F**). Specifically, fewer TRAP^+^ OC cells were observed in the 10 μM GA-treated groups.

### GA suppresses osteoclast formation in a time-dependent manner

**Figure 2:**
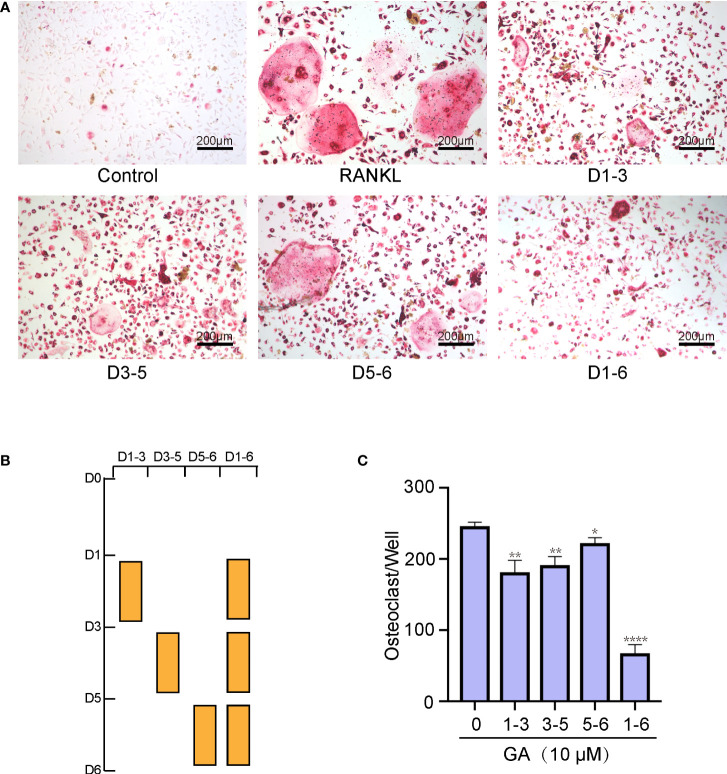
GA suppresses osteoclast formation in a time‐dependent manner. **(A, B)** Representative images of the TRAP-positive cells treated with 10 μM GA on days 1–3, 3–5, 5–6, and 1–6. **(C)** Quantitative analysis of the number of TRAP-positive multinucleated OCs (>3 nuclei) at different time points. Scale bar = 200 μm. n= 5, *P < 0.05, **P < 0.01, ****P < 0.0001 versus RANKL-induced group.

To determine which time stage of OC differentiation was influenced, BMMs were intervened with 10 μM GA on the groups of days 1–3, 3–5, 5–6, and 1–6. As shown in **Figures 2A, B**, GA had a remarkable inhibitory effect in all stages of OC differentiation, whereas the effects were weakened when GA was added on day 5‐6, revealing that GA inhibits OC differentiation mainly at the early (day 1–3) and middle (day 3–5) stages. Obviously, the number and size of multinucleated TRAP^+^ OC in the group of day 1-6 was significantly lower than that in the control group (**Figure 2C**).

### GA reduces F-actin formation and osteoclast resorptive activity

**Figure 3:**
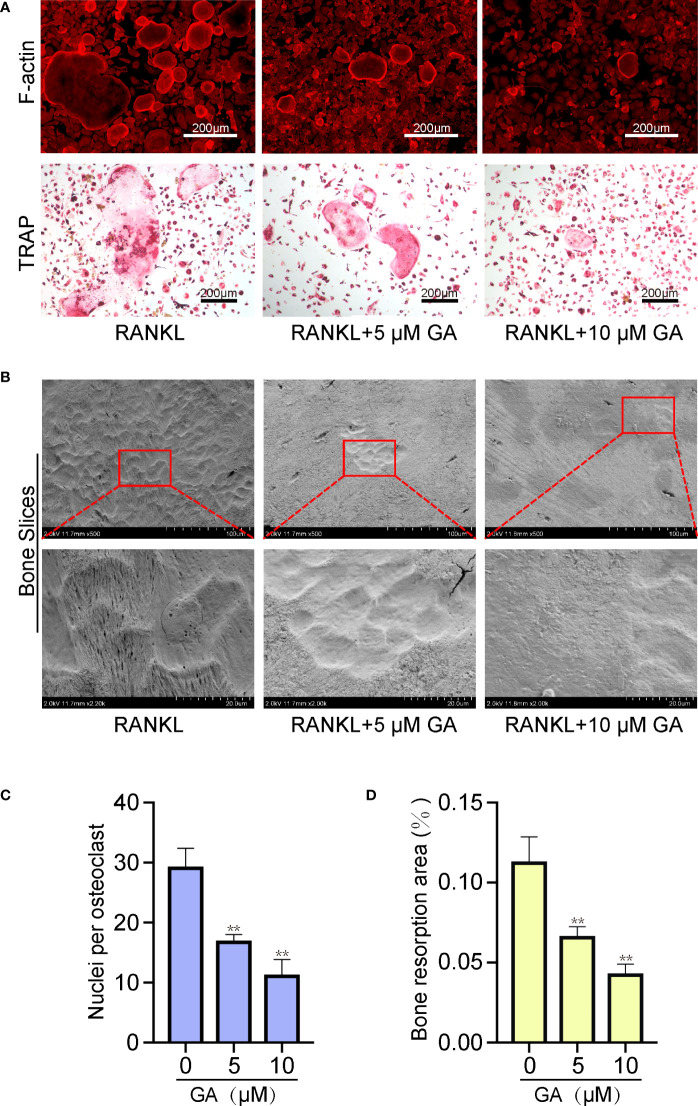
GA reduces F-actin formation and osteoclast resorptive activity. **(A)** Representative images of fluorescence staining and TRAP staining of OC treated without or with GA (5 and 10 μM). Scale bar = 200 μm. **(B)** Representative images of the pit formation on bone slice without or with GA treatment. **(C)** Analysis of the number of OC nuclei by TRAP staining. **(D)** Quantitative analysis of the bone resorption areas on bone slices. n= 5, **P < 0.01 versus RANKL-induced group.

Additionally, the fluorescence staining of cytoskeleton was performed to examine the effect of GA on OC fusion. **Figures 3A, C** show that the average area of each F-actin belt formation and the number of nuclei of each OC by TRAP staining were markedly decreased with 5 and 10 μM GA.

Beyond the formation and fusion of OC, we further tested the ability of GA to attenuate the osteoclastic resorptive activity using bone slices. After incubating with RANKL (50 ng/ml) in the presence or absence of GA (5 μM, 10 μM) for 10 days, the resorption pits were observed by scanning electron microscopy and measured by ImageJ. **Figures 3B, D** showed that the bone resorption area obviously reduced as the drug concentration increased, especially at 10 μM, suggesting that GA can suppress the resorptive activity of OC.

### GA inhibits relevant osteoclastic genes and proteins expression

**Figure 4:**
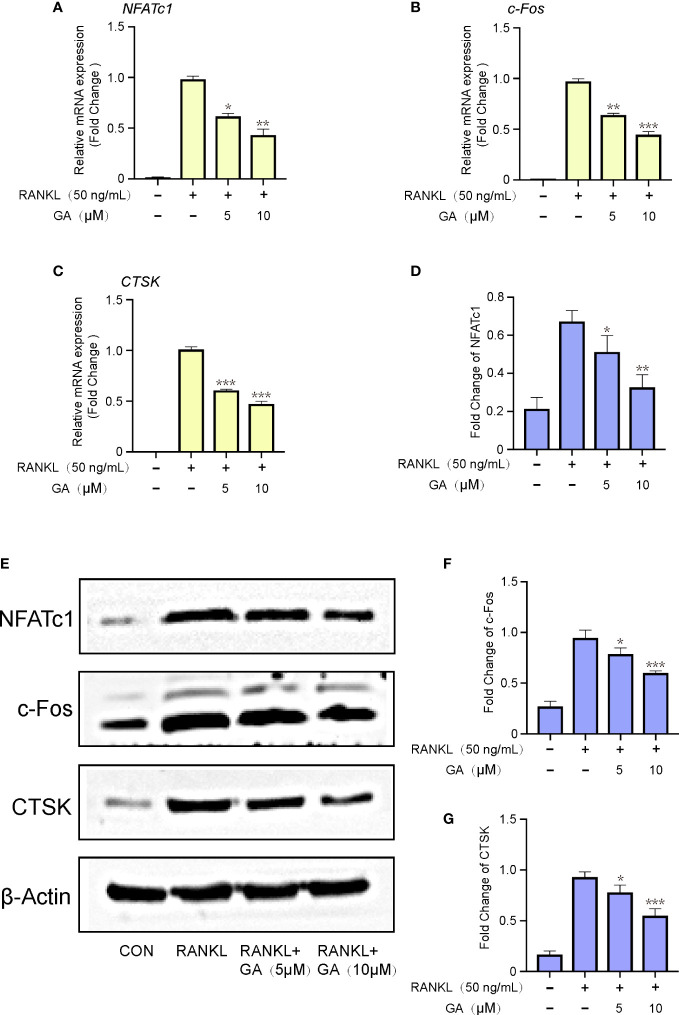
GA inhibits relevant osteoclastic genes and proteins expression. **(A-C)** The gene expression of NFATc1, c‐Fos, CTSK after stimulation by RANKL with or without GA (5 and 10 μM). The expression of target genes was normalized to β-actin. **(D)** The protein expression of NFATc1, c‐Fos, CTSK after stimulation by RANKL with or without GA (5 and 10 μM). **(E-G)** The expression of target proteins was normalized to β‐actin. n= 3, *P < 0.05, **P < 0.01, and ***P < 0.001 versus RANKL‐induced group.

Next, a qRT‐PCR assay of relevant osteoclastic genes, including NFATc1, c-Fos, and CTSK, were dose-dependently down-regulated by GA treatment (5 and 10 μM). The expression of these target genes in the RANKL group were remarkably elevated than that in the control group, while obviously downregulated by GA treatment (**Figures 4A–C**). Meanwhile, the results of western blot also demonstrated that GA can effectively inhibited the expression of these osteoclastic proteins (**Figures 4D–G**).

### GA represses RANKL-induced ERK, JNK and AKT signaling pathways

**Figure 5:**
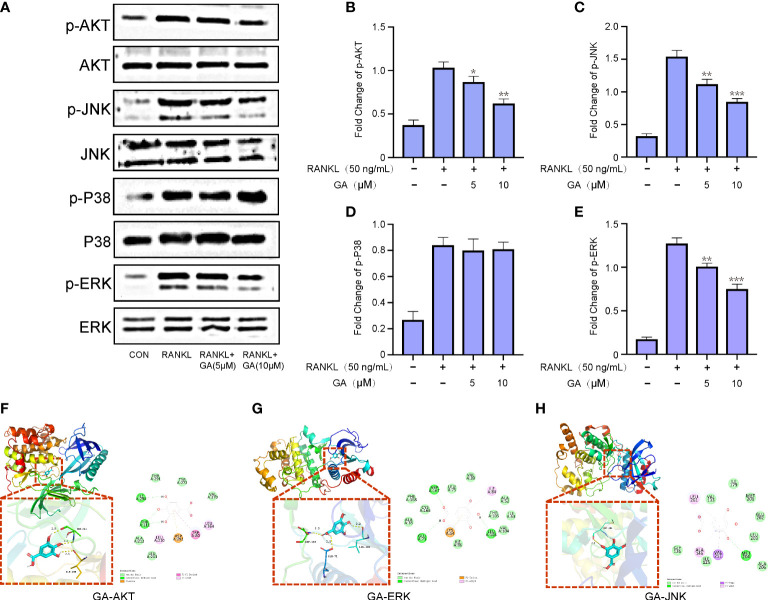
GA represses RANKL-induced ERK, JNK and AKT signaling pathways. **(A)** Western blot analysis of p-AKT, p-JNK, p-P38 and p-ERK in BMMs treated with RANKL in the presence or absence of GA (5 and 10 μM). **(B-E)** The relative ratios of phosphorylation levels of proteins to total protein levels were quantified. n= 3. **(F-H)** The free binding energy for molecular docking of GA and ERK, Akt and JNK. *P < 0.05, **P < 0.01, ***P < 0.001 versus RANKL‐induced group.

To delve into the mechanisms involved in the suppressive effect of GA on OC formation, western blot was further performed to discover whichever pathway was blocked. BMMs were induced with RANKL and M-CSF stimulation and GA (5, 10 μM) intervention for 1 h, and then the Akt and MAPK pathways were detected (**Figure 5A**). As depicted in **Figures 5B–E**, the expression levels of p-AKT, p-JNK and p-ERK were dose-dependently repressed by GA, whereas the p-P38 wasn’t significantly different between the groups with and without GA. To further confirm that GA can act on ERK, Akt and JNK, we constructed molecular docking for GA-ERK, GA-Akt and GA-JNK. The free binding energy of GA with Akt, ERK, and JNK were -6.0 kcal/mol, -6.1 kcal/mol, and -5.7 kcal/mol, respectively, revealed that Akt, ERK, and JNK had a strong affinity for GA (**Figures 5F–H**).

### GA protects against OVX-induced bone loss *in vivo*

**Figure 6:**
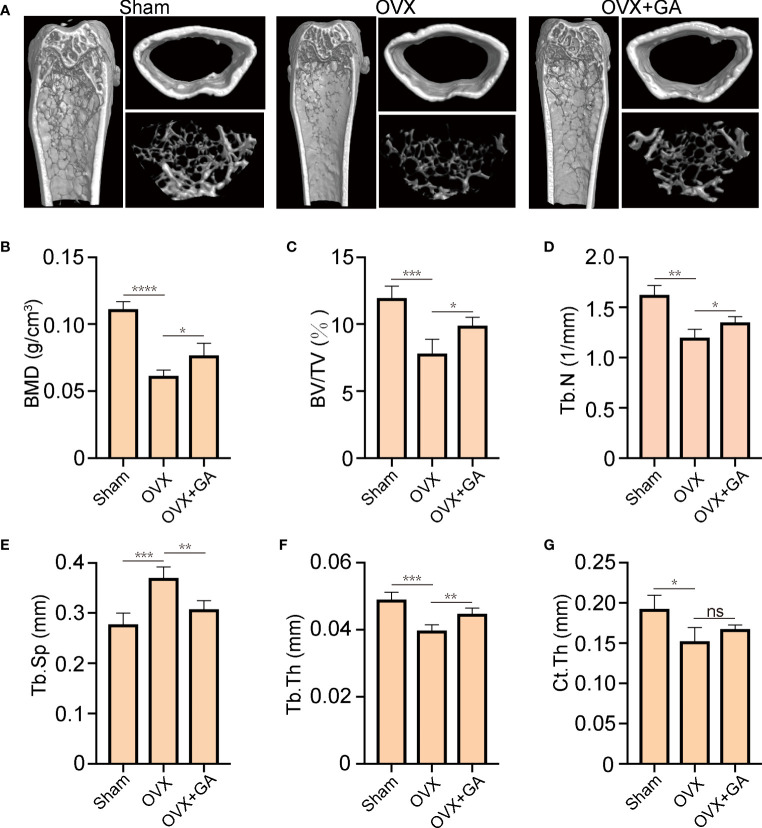
GA protects against OVX-induced bone loss *in vivo*. **(A)** Representative 3D reconstructed CT images of the distal femur microstructure. **(B‐G)** Quantitative analyses of BMD, BV/TV, Tb.N, Tb.Th, Tb.Sp, and Ct.Th in the groups. n= 7, *P < 0.05, **P < 0.01, ***P < 0.001, and ****P < 0.0001. ns, non-significant.

To further evaluate the actual effect of GA, an OVX mice model was established and treated with GA. Subsequently, Micro‐CT were performed and the following parameters were analyzed (**Figure 6A**). Compared with the OVX group, the following parameters of BMD, BV/TV, Tb/Th and Tb.N of GA-treated group were obviously increased, while the trabecular spacing (Tb.Sp) was decreased (**Figures 6B–F**). However, Ct.Th remained unchanged between the OVX group and GA-treated group in the current study (**Figure 6G**).

**Figure 7:**
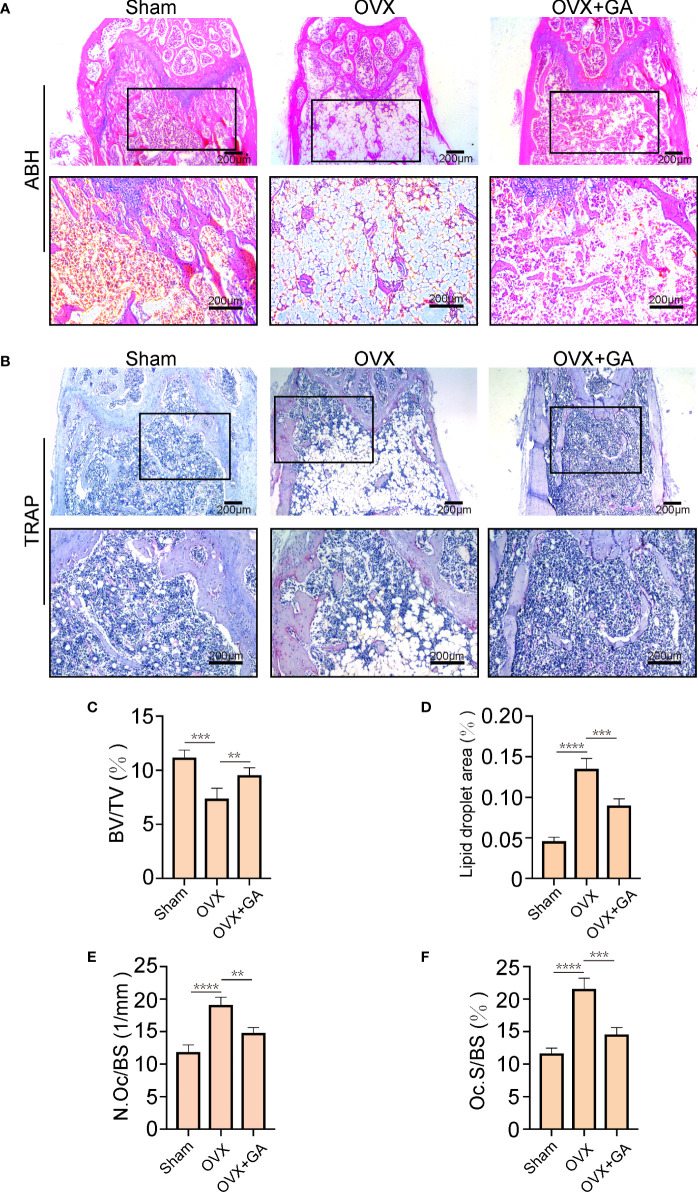
GA ameliorates OVX-induced bone loss by inhibiting osteoclast activity *in vivo*. **(A, B)** Representative images of decalcified bone stained with ABH and TRAP from mice in the sham, OVX, and GA (10 mg/kg) treatment groups. Scale bar = 200 μm. **(C-F)** Quantitative analyses of BV/TV, area of lipid droplets, and TRAP-positive cells. n= 7, **P < 0.01, ***P < 0.001, ****P < 0.0001.

Histomorphometric analyses was further applied to observe the alterations in bone microstructure. The results of ABH staining indicated that the BV/TV were remarkably lower in the OVX group than that in the sham group, accompanied by a large amount of fat accumulation. Interestingly, both the BV/TV and fat accumulation were significantly improved after GA treatment (**Figures 7A, C, D**). Moreover, TRAP staining showed that GA-treated group had fewer TRAP^+^ OC than those from OVX group, with decreased Oc.S/BS and the N.Oc/BS (**Figures 7B, E, F**).

### GA decreases the expression of osteoclast-associated protein *in vivo*

**Figure 8:**
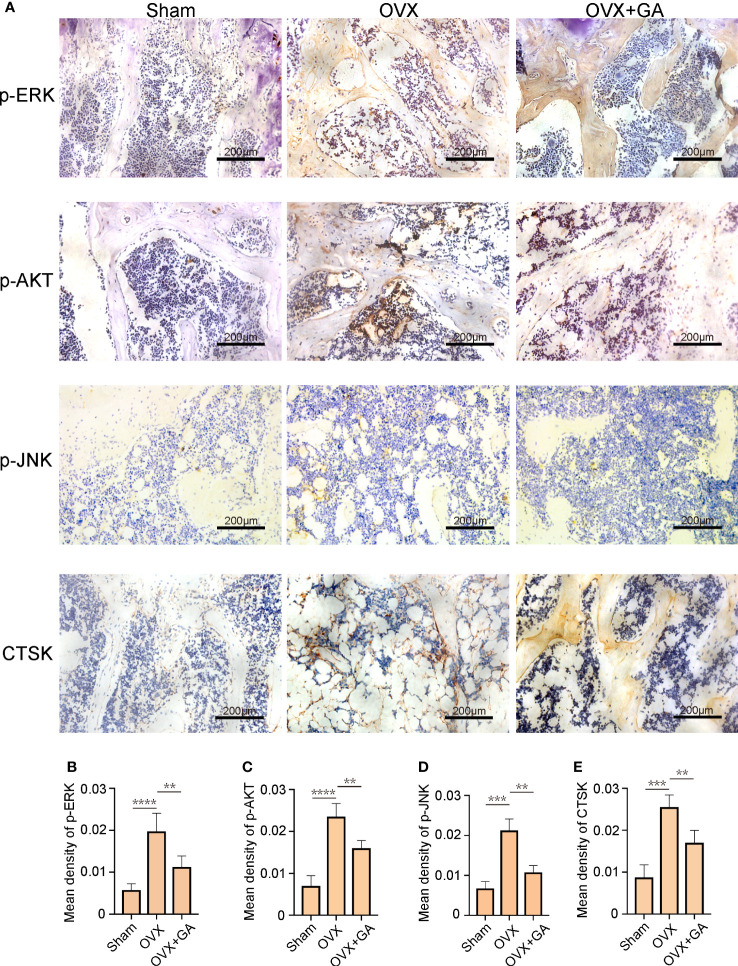
GA decreases the expression of osteoclast-associated protein *in vivo.* **(A)** Immunohistochemical staining for p-ERK, p-Akt, p-JNK, and CTSK in the sham, OVX, and OVX+GA groups. Scale bar = 200 μm. **(B-E)** Quantitative analysis of the intensity of immunostaining for p-ERK, p-Akt, p-JNK, and CTSK. n= 7, **P < 0.01, ***P < 0.001, ****P < 0.0001.

Furthermore, to explore the effect of GA on the osteoclast-associated protein expression (p-ERK, p-Akt, p-JNK, and CTSK) *in vivo*, we performed IHC assay to detect the expression of p-ERK, p-Akt, p-JNK, and CTSK. The results of IHC assay showed that the expression of p-ERK, p-Akt, p-JNK, and CTSK in the OVX group were obviously increased than that of the sham group, while these changes were reversed by GA treatment (**Figure 8A**). It was evident that the GA-treated group showed a significant reduction in these proteins by quantitative analysis (**Figures 8B–E**).

## Discussion

In the current study, we found that GA exerts notable effects in inhibiting osteoclastogenesis and preventing ovariectomy-induced bone loss. The results of *in vitro* experiments showed that GA inhibited OC formation and function by affecting related genes and proteins through suppressing the AKT and ERK and JNK signaling pathways. Moreover, histological and immunohistochemical results showed that GA protects against OVX-induced bone loss *in vivo*.

Since excessive activation of OC plays a dominant role in bone loss, the development of new drugs to inhibit OC differentiation is a major and promising target for the treatment of osteoporosis (20, 21). However, the clinical antiresorptive agents for osteoporosis are accompanied by several side effects, including osteonecrosis of the jaw, gastrointestinal discomfort, and hypokalaemia (22), new therapeutic strategies to prevent osteoporosis are urgently needed. In recent years, several studies have been reported the therapeutic effects of natural compounds on osteoporosis due to their wide range of biological activities and fewer side effects (23–25). Similarly, our study found that GA also showed the potential value in the treatment of osteoporosis.

Multinucleated OC cells are formed by the fusion of multiple BMMs, and the fusion process is regulated by two essential cytokines (M-CSF and RANKL). During OC formation and maturation, NFATc1 and c‐Fos are two indispensable transcription factors that activate a series of genes participated in OC formation (26). OC mainly performs the function of bone resorption, when OC attaches to the bone surface, it will secrete numerous enzymes to accomplish bone absorption, such as CTSK and TRAP. Among them, CTSK plays a pivotal role in resorption of bone matrix, while TRAP can enhance CTSK activity (27). In our research, the expression levels of NFATc1, c‐Fos, and CTSK were obviously decreases by GA treatment. Consistently, TRAP staining results showed that the number of TRAP^+^ OC was significantly reduced with GA treatment. Our results indicate that GA has an inhibitory effect on the differentiation and function of OC.

During OC formation and maturation, the binding of RANKL to its receptor RANK activates a cascade of downstream signaling pathways, such as Akt and MAPK, which have been shown to regulate the process of osteoclastogenesis (28, 29). Accumulated evidence indicated Akt was activated by M-CSF and RANKL, and served as a central player in the regulation of osteoclast survival and differentiation (30, 31). The activation of Akt stimulates osteoclastogenesis by mediating the GSK3β/NFATc1 signaling cascade (32). In our study, GA effectively suppressed RANKL-induced osteoclastogenesis by inhibiting AKT phosphorylation. Meanwhile, MAPK, including ERK, JNK, and P38, is another important signaling pathway in osteoclast differentiation. It has been reported that taking appropriate measures to interrupt the phosphorylation of p38, JNK and ERK can inhibit osteoclast formation (33–35). According to our results, GA markedly inhibited p-AKT, p-ERK and p-JNK expression at 1 h after RANKL stimulation, whereas it has no effect on p-P38 expression. These findings indicate that GA downregulates the expression of OC-related gene and protein by interfering with the upstream AKT, ERK and JNK pathways, ultimately resulting in suppressed osteoclast differentiation.

Estrogen can block OC differentiation; Conversely, estrogen withdrawal dramatically promotes OC formation and facilitates bone resorption (36). Thus, we further verify the preventive effect of GA on bone loss by using an OVX-induced osteoporosis mice model. The results of micro-CT analysis and histological examination suggested that GA effectively improve the trabecular microarchitecture *in vivo*, as relatively slight bone loss was observed by GA treatment. Besides, immunohistochemistry assay showed that the levels of p-AKT, p-ERK, p-JNK and CTSK expression was obviously reduced in GA group, which is consistent with the *in vitro* experimental results.

In summary, our study indicated that GA exerts notable effects in suppressing osteoclastogenesis and preventing ovariectomy-induced bone loss through inhibition of Akt, JNK and ERK signaling pathways, suggesting that GA is a potential agent in osteoporosis treatment.

## Data availability statement

The raw data supporting the conclusions of this article will be made available by the authors, without undue reservation.

## Ethics statement

The animal study was reviewed and approved by The Ethics of Animal Experiments of Zhejiang Chinese Medical University.

## Author contributions

PZ wrote the first draft of the manuscript. JY and JD contributed to conception. YW and GC organized the database. JH performed the statistical analysis. QH and JF designed the study. All authors contributed to the article and approved the submitted version.
